# Hydrophilic and Hydrophobic: Modified GeO_2_ Aerogels by Ambient Pressure Drying

**DOI:** 10.3390/nano14181511

**Published:** 2024-09-18

**Authors:** Varvara O. Veselova, Sergey Yu. Kottsov, Svetlana V. Golodukhina, Daria A. Khvoshchevskaya, Olga M. Gajtko

**Affiliations:** 1 Kurnakov Institute of General and Inorganic Chemistry, Russian Academy of Sciences, Leninsky Prospect 31, Moscow 119991, Russia; sergey12-17@yandex.ru (S.Y.K.);; 2 Faculty of Materials Science, Lomonosov Moscow State University, Leninskie Gory 1, Building 73, Moscow 119991, Russia

**Keywords:** aerogel, germania, surface, modification, ambient pressure drying, supercritical drying

## Abstract

An ever-increasing number of applications of oxide aerogels places a high demand on wettability-tuning techniques. This work explores the possibility to cheaply prepare GeO_2_ aerogels with controlled wettability by an ambient pressure drying (APD) method. GeO_2_ aerogels are prepared via two synthetic routes. Surface modification is carried out by soaking the gels in a silylating agent solution; type and concentration of the modifier are optimized to achieve a large surface area. The aerogels have been characterized by Fourier transform infrared spectroscopy, scanning electron microscopy, nitrogen adsorption and contact angle measurements. The effect of surface modification on the phase composition and particle size of the aerogels is described. In summary, the work provides a new cheap production method for the preparation of both hydrophobic and hydrophilic GeO_2_ aerogels with contact angle varying from 30° to 141° and with surface area of 90–140 m^2^/g, which facilitates the expansion of their diverse applications. GeO_2_ aerogel synthesis by APD is reported for the first time.

## 1. Introduction

The use of various compounds in the form of aerogel allows us to achieve significant advantages. For example, aerogels have increased catalytic activity [1,2]; aerogel-based LIB electrodes are more stable compared to bulk materials [3,4]; aerogels have improved spectral and kinetic characteristics of luminescence [5,6,7]. Aerogels have found multiple applications in both science and commercial technology.

However, the large-scale production of aerogels is limited due to the complex and long stage of supercritical drying (SCD). Drying at atmospheric pressure (ambient pressure drying, APD) without reducing the specific surface area of the material and maintaining the porous structure is possible, but it requires chemical modification of the gel surface with hydrophobic compounds [8]. Such modification makes it possible to avoid pore collapse during evaporation of the solvent.

In addition, a common obstacle to the wider use of these materials is their sensitivity to moisture. This is due to the presence of polar –OH groups on the surface of the aerogel. These groups participate in the adsorption of water from the atmosphere, which eventually leads to the degradation of the material. Chemical hydrophobization of the aerogel helps to avoid this problem as well.

The approaches to aerogel surface modification are studied extensively for but also the specific surface area and pore structure of the material [9,10,11]. To successfully produce an aerogel via the APD route it is necessary to use a silylating agent that is suitable for specific surface groups on the gel nanoparticles and for the synthesis conditions.

Despite the similar chemical properties of silicon and germanium, attempts to obtain aerogels based on pure GeO_2_ are extremely few [12,13,14], and to the best of our knowledge there is no work on modifying its surface and producing GeO_2_ via APD. This topic remains virtually unexplored, despite GeO_2_ aerogel exhibiting very unusual luminescence [13]. Nanostructured GeO_2_ materials also draw huge interest as LIA anode material [15]. In the present work, the possibility of obtaining GeO_2_ aerogels by APD was studied. Gels with different preprocessing history were used to estimate the role of synthesis conditions, solvent etc. on the modification process. In the first method the starting lyogel was obtained using an epoxy-induced sol–gel process in the GeCl_4_-ButAc-propylene oxide-HCl system (Gel-1). Another gel was formed in the GeO_2_-NH_3_-H_2_O system (Gel-2, which is described in more detail in [16,17]).

Methyltrimethoxysilane (MTMS), dimethylchlorosilane (DMCS) and hexamethyldisilazane (HMDS) were selected as silylating agents with different hydrolytically active sites for gels with different histories. The work is supposed to test how these modifiers would affect the physico-chemical properties of aerogels dried at atmospheric pressure. The chemical modification of the surface was carried out by postsynthetic treatment, namely by soaking the gels in a silylating agent solution.

For both of the obtained gels the optimal solvent, modifier and concentration of the modifier were determined. The optimized conditions allowed us to obtain GeO_2_ aerogels by APD with surface area of 90–140 m^2^/g. It was demonstrated that, depending on the synthesis conditions, both hydrophilic and hydrophobic GeO_2_ aerogels can be prepared by APD.

## 2. Materials and Methods

The following reagents were used: germanium oxide GeO_2_ (99.999%, Acros, Geel, Belgium), GeCl_4_ (99.99%, Sigma-Aldrich, St. Louis, MO, USA), aqueous ammonia (25%, Aldosa, Podolsk, Russia), heptane (99%, Himmed, Moscow, Russia), dimethylformamide (99.9%, Himmed, Moscow, Russia), butyl acetate (reagent grade, Himmed, Moscow, Russia), propylene oxide (99.5%, Sigma-Aldrich, St. Louis, MO, USA), hydrochloric acid (puriss. spec., Sigma Tech, Moscow, Russia), methyltrimethoxysilane (MTMS) (95+%, Merck, Darmstadt, Germany), dimethylchlorosilane (DMCS) (99.5%, Sigma-Aldrich, St. Louis, MO, USA) and hexamethyldisilazane (HMDS) (99%, Sigma-Aldrich, St. Louis, MO, USA), distilled water.

The initial gels were obtained using the following techniques:

(Gel-1) To obtain a gel, 0.1 mL (0.87 mmol) of GeCl_4_ was dissolved in 2.294 mL (17.4 mmol) of butyl acetate, 0.609 mL (8.7 mmol) of propylene oxide was added to the solution and the resulting solution was cooled to −18 °C. The obtained solution was placed in a polypropylene container and 0.081 mL of hydrochloric acid (4.35 mmol) was added under ultrasonic exposure. The molar ratio of reagents in the system of GeCl_4_:ButAc:HCl:Propylene oxide is 1:20:5:10. The gel formed within two minutes. The volume of the resulting gel was 3 mL. The obtained gel was placed in 6 mL of heptane solution with modifier content from 1 to 15 wt.% for 1 day. Then the modifier solution was replaced with a pure solvent once a day for 2 days before drying at 50 °C for 12 h.

(Gel-2) To obtain a gel, 0.3154 g of GeO_2_ (3.015 mmol) was suspended in 4.625 mL of distilled water with constant stirring on a magnetic stirrer (IKA, Staufen, Germany) , then 375 µL of 25% ammonia solution was added. The molar ratio of germanium oxide to ammonia was 3:5. The resulting suspension was stirred for 24 h. The formed sol (pH ~10.4) was transferred to cylindrical polypropylene containers (5–10 mL). Gelation took place within two days. The volume of the resulting gel was 5 mL. The obtained gel was placed in 6 mL of DMF solution with modifier content from 1 to 15 wt.% for 1 day. Then the modifier solution was replaced with a pure solvent once a day for 2 days before drying at 50 °C for 12 h. The initial samples obtained had the composition (NH_4_)_3_H(Ge_7_O_16_)(H_2_O)_x_ and were additionally annealed at 300 °C to produce GeO_2_ aerogels.

To prepare control samples for comparison the obtained gels without surface modification were dried in supercritical CO_2_ (t_cr_ = 31°C, P_cr_ = 72.8 atm). Gel-1 was dried as-is, and Gel-2 underwent a solvent exchange procedure to replace water with DMF. The SCD was carried out in an installation consisting of a Supercritical 24 high-pressure CO_2_ pump (SSI, Chicago, IL, USA), a steel reactor with a capacity of 50 mL and a back pressure regulator (BPR) (Goregulator, Waters, Milford, MA, USA). The sample was washed with liquid CO_2_ for 2 h at a temperature of 20 °C and a pressure of 15 MPa, then the temperature in the reactor was increased to 50 °C and the sample was washed with supercritical CO_2_ (15 MPa) for 2–2.5 h. Then, gradually (for 30–40 min), the pressure in the heated autoclave was reduced to atmospheric, after which the autoclave was cooled and opened.

The Bruker (Billerica, MA, USA) D8 Advance X-ray diffractometer (CuKα radiation, Ni filter and Lynxeye detector) was used to identify the phase composition of the product. Diffraction data were collected in the range of 2θ from 7° to 65° with a step of 0.02° and accumulation time of 0.3 s/step. The identification of diffraction maxima was carried out by comparison with the JCPDS database. The size of the coherent scattering region was estimated using the Scherer formula.

The morphology of the obtained samples was studied by scanning electron microscopy using the Tescan Amber GMH high-resolution electron microscope (Tescan Orsay Holding, a.s., Brno–Kohoutovice, Czech Republic) at an accelerating voltage of 1 kV using an Everhart–Thornley detector. To prevent image artifacts, the samples were analyzed without the deposition of any conducting layer on their surface. Processing of the microphotographs was carried out using Gwyddion 2.66 free data analysis software.

Energy-dispersive X-ray microanalysis (EDX) was performed on a high-resolution Tescan Amber GMH electron microscope using an integrated energy-dispersive spectroscopy (EDS) detector (Oxford Instruments (Abingdon, UK) Ultim Max 100 mm^2^) at an accelerating voltage of 20 kV, and the probe current was calibrated on the cobalt standard.

Diffuse reflection IR spectra were recorded using a laboratory IR Fourier spectrometer FSM 2202 (Infraspek Ltd., Saint-Petersburg, Russia) relative to KBr in the range of 400–4000 cm^−1^ with a resolution of 2 cm^−1^, averaging 100 spectra. To register the spectra, the samples were diluted in potassium bromide to a mass fraction of 3%. GeO_2_ (99.99%, Acros, Geel, Belgium) was used to register the IR spectra of crystalline germanium oxide.

Low-temperature nitrogen adsorption experiments were conducted using a Katakon (Novosibirsk, Russia) Sorbtometer-M low-temperature nitrogen adsorption system. Prior to analysis, the samples were degassed in a dry helium flow at 80 °C for 2 h. For the specific surface area (SBET) calculation, 5 experimental points were measured in the partial pressure range of 0.05–0.25 P/Po and the Brunauer–Emmett–Teller (BET) model was applied. Full nitrogen adsorption–desorption isotherms were measured using the same system in a P/Po range of 0.01–0.97. The pore size distributions were constructed within the Barett–Joyner–Halenda (BJH) model using the desorption branch of the isotherm. The PyGAPS 4.5.0 software package was used to process the experimental data [18].

The contact angles were measured on an FT A 200 device (First Ten Angstroms, Inc., Portsmouth, VA, USA). The resulting photos were processed using FT200 software.

## 3. Results and Discussion

### 3.1. Choice of Modifying Agent and Solvent for Gels with Different Prehistory

Gels obtained by two different methods were used. This approach was chosen because a huge effect of synthetic route on the surface chemistry was shown in [19] for photoluminescent GeO_2_ nanoparticles. Thus, two different gels were used to establish the role of gel preprocessing history on the surface modification process. The development of the gel synthesis procedures is described elsewhere [16,17] and is not the focus of the present work. Synthesis details are provided in the experimental sections. Further in the text the used gels will be referred to as Gel-1 and Gel-2. The significant differences of these gels’ synthesis parameters are summarized in Table 1.

An important stage of the work was the selection of a solvent for gels with different backgrounds. The solvent had to meet the following requirements: (a) dissolve the modifiers, (b) not react with the modifiers and (c) maintain the integrity of the gels. Straight-chain liquid hydrocarbons are most commonly used for such surface modification. However, Gel-2 turned out to be unstable in such solvents—the aquagel quickly disintegrated and formed a sol. As a result, heptane was selected for Gel-1 and DMF for Gel-2 [20] (Table 1).

**Figure 1:**
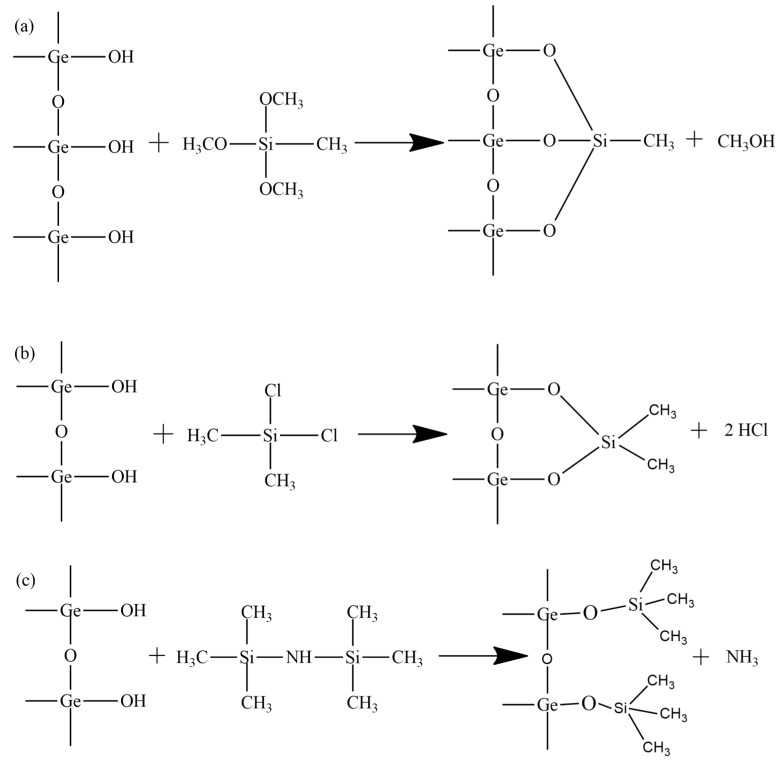
Hydrolytically active sites and resulting surface groups for: (**a**) MTMS, (**b**) DMCS, (**c**) HMDS.

To carry out the modification, it is necessary to use a silylating agent that is suitable for specific surface groups. That is, for gels with different histories the modifier must be selected individually. Silylating agents such as hexamethyldisilazane (HMDS), methyltrimethoxysilane (MTMS) and dimethylchlorosilane (DMCS) were used as starting materials, as it was previously shown that they are among the most suitable for drying at atmospheric pressure [21,22]. These reagents have different hydrolytically active sites (Figure 1), which means they would react differently depending on the pH, solvent and surface chemistry of the nanoparticles.

It was previously reported [10] that the optimal content of silylating agents lies in the range of 10–20 wt.%. Initial experiments on the selection of a suitable modifier were carried out using 10 wt.% solutions of silylating agents (Table 2). For Gel-1 the largest specific surface area was achieved with the use of methyltrimethoxysilane (MTMS). For Gel-2 the largest S_BET_ was obtained with hexamethyldisilazane (HMDS).

It is interesting that the largest surface area in the case of Gel-2 was obtained by using HMDS for modification. This agrees well with results obtained for waterglass-based SiO_2_ aerogels as well [23]. In the cited work HMDS was used for surface modification in hexane in the presence of nitric acid. In our case this modifier was shown to be most effective for a similar waterglass-like system with DMF as solvent at basic pH. This leads to the conclusion that in this particular case nanoparticle surface groups play a key role in the interaction.

After determining the most suitable silylating agent, it was necessary to select the optimal content of the modifier, at which the greatest specific surface area would be reached. Solutions with a modifier content from 1 to 15 wt.% of the silylating agent were tested. Varying the concentration of the modifier showed that for both initial gels, the largest specific surface area of aerogels was achieved with a 5 wt.% content of the silylating agent in the solution (Table 3). Surface area of aerogels obtained by supercritical drying of the same gels is given as a reference.

The ratio of germanium in the gel to silylating agent in the solution varied in a wide range and is given in Table 3 for reference. However, not all the silylating agent present in the solution interacts with the gel surface. According to EDX data, in dried Gel-1 treated with 5 wt.% MTMS solution the Ge/Si ratio is 2.4 (while the ratio of Ge atoms in the gel to Si atoms present in the modifying agent solution was 0.1, as shown in Table 3) which means that approximately 1/25 or 4% of the silylating agent molecules were bonded to the gel matrix. EDX mapping showed very uniform distribution of silicon atoms in the aerogel, with average deviation of only 1at.%.

In the case of Gel-2 the content of silicon is at the limit of the EDX method’s sensitivity. However, it is evident that the modifier does interact with the matrix, since the surface area depends on its content, as shown in Table 3.

### 3.2. Characterization of Gel-1

**Figure 2:**
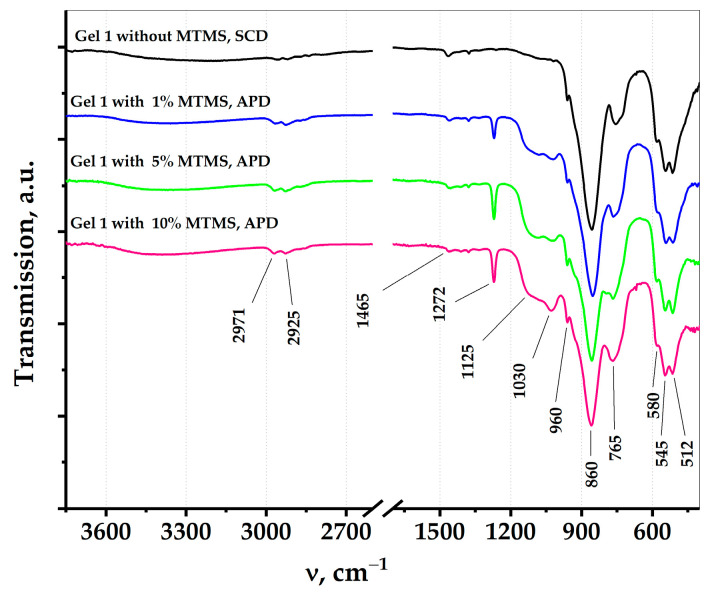
IR spectra of the aerogels obtained using supercritical drying and aerogels obtained by drying at atmospheric pressure (with modifier, MTMS).

The surface modification was confirmed by comparing the IR spectra of aerogels prepared by SCD and by APD (Figure 2). In the IR spectra of all the samples the bands associated with the vibrations of germanium atoms are present: deformation vibrations of the Ge–O–Ge groups at 500–590 cm^−1^ [24], the asymmetric vibrations of Ge–O– bonds at 765 cm^−1^ and two different modes of the Ge–O–Ge asymmetric stretching vibrations at 860 cm^−1^ and 960 cm^−1^ [14,25]. The low-intensity bands at 2800–3010 and 1465 cm^−1^ could be attributed to the C–H symmetric and asymmetric stretching vibration of –CH_3_, –CH_2_ and –CH groups and the deformation vibration of C–H, respectively [26]. Taking into account that these bands are also observed in the spectrum of the unmodified aerogels, they must have originated from propylene oxide residues, but not from MTMS. Three bands (1030, 1125 and 1272 cm^−1^) are characteristic only for aerogels modified with MTMS. The band at 1125 cm^−1^ is ascribed to the asymmetric stretching of the Si–O bonds, at 1029 cm^−1^ to the asymmetric stretching vibrations of the Si–O–Si bonds and at 1272 cm^−1^ to the asymmetric stretching of the Si–C bonds [27,28].

**Figure 3:**
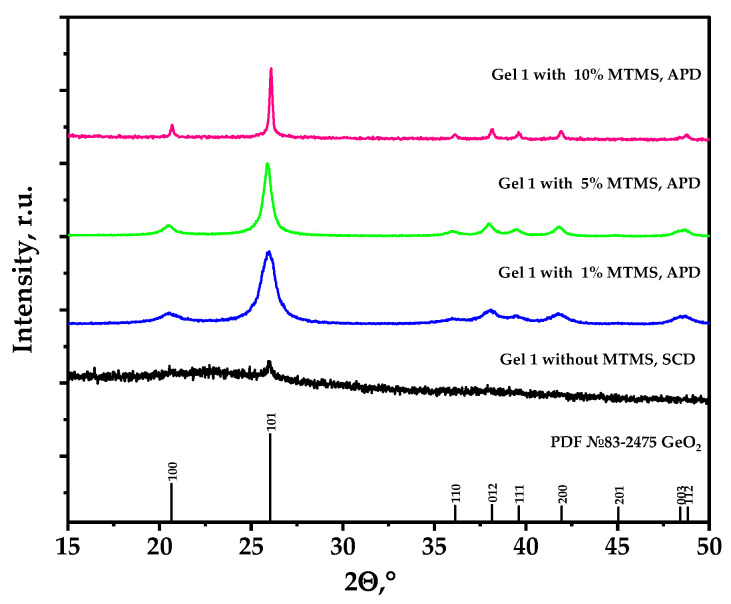
XRD patterns of aerogel obtained using supercritical drying and aerogels obtained by drying at atmospheric pressure (with modifier, MTMS).

Samples of the gels obtained by the epoxide-induced sol–gel process and dried in supercritical CO_2_ were predominantly amorphous. However, the introduction of MTMS and drying at atmospheric pressure led to the formation of crystalline aerogels, and with an increase in the content of MTMS, the degree of crystallinity increased (Figure 3). The CSR calculated by the Scherrer equation is 8 nm at 1% MTMS content, 16 nm at 5% MTMS content and reaches 50 nm at 10% MTMS.

**Figure 4:**
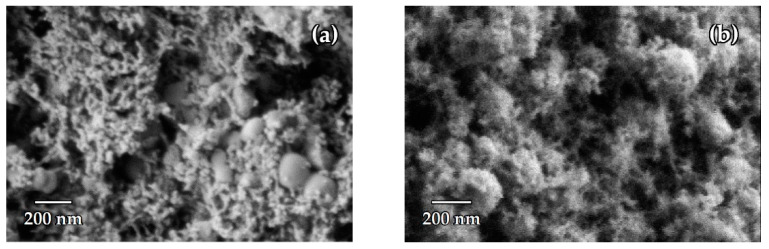
SEM images of aerogel obtained by drying Gel-1 in supercritical CO_2_ (**a**) and by drying Gel-1 modified with 5 wt.% MTMS at atmospheric pressure (**b**).

Microphotographs of the aerogels obtained by SCD and APD evidence that in both cases the samples consist of bigger spherical aggregates covered by a nanosized 3D network (Figure 4). That is, the modification process does not significantly alter the morphology of the product, just the phase composition (Figure 3). In the case of the SCD dried aerogel the size of the agglomerates estimated from the SEM images is 100–200 nm, and in the case of APD it is 100–300 nm.

**Figure 5:**
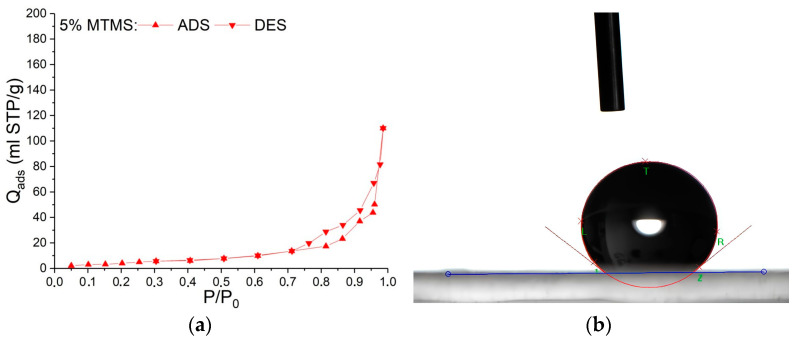
Isotherms of low-temperature adsorption–desorption of nitrogen in aerogel obtained by drying Gel-1 modified with 5 wt.% MTMS at atmospheric pressure (**a**); Photo of a drop on the surface of the aerogel obtained by drying Gel-1 modified with 5 wt.% MTMS at atmospheric pressure, contact angle 141° (**b**).

Nitrogen adsorption–desorption isotherms of aerogel obtained by drying Gel-1 modified with 5 wt.% MTMS at atmospheric pressure belong to Type III according to IUPAC, which is typical for macroporous materials, with H3 type hysteresis (Figure 5a). The Barrett–Joyner–Halenda model is not applicable for this type of isotherm, so the average pore size and the cumulative pore volume could not be calculated for this sample [29].

For the aerogel obtained by APD with maximum surface area (5 wt.% modifier), contact wetting angle was determined. This aerogel exhibited hydrophobic properties and the contact angle was 141° (Figure 5b). Without a modifier all the samples obtained in SC conditions exhibited hydrophilic properties, and the contact wetting angle of this aerogel was 39° for Gel-1.

### 3.3. Characterization of Gel-2

**Figure 6:**
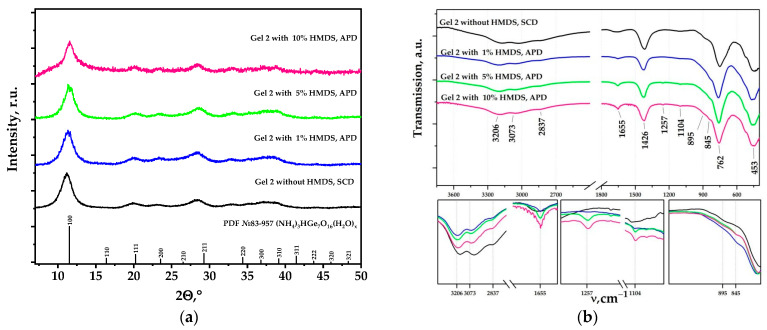
Diffractograms (**a**) and IR spectra (**b**) of aerogel obtained using supercritical drying and aerogels obtained by drying at atmospheric pressure (with modifier, HMDS).

Samples of the (NH_4_)_3_H(Ge_7_O_16_)(H_2_O)_x_ composition were obtained in the GeO_2_-NH_3_-H_2_O system, regardless of the drying method and modifier content (Figure 6a). No correlation of the phase composition and particle size with the content of the modifier was observed for this system.

There are no such significant changes in the IR spectra of aerogels obtained using Gel-2 as a precursor as in the case of Gel-1. Nevertheless, in addition to the bands related to vibrations of the germanate lattice (453, 762 and 895 cm^−1^) [24] and ammonium group (1426 cm^−1^) [30], there are very weak bands at 845 (as shoulder), 1104 and 1257 cm^−1^ which increase in intensity with the increasing HMDS concentration (Figure 6b). These bands could be attributed to the asymmetric stretching vibration of Si–O group (1104 cm^−1^) and Si–CH_3_ deformation (845 and 1257 cm^−1^) [31,32]. There is also an increase in the intensity of the band at 1655 cm^−1^, which is associated with the superposition of the Si–OH vibration with the vibrations of water molecules. The large halo in the high-frequency region (2500–3600 cm^−1^) is attributed to the hydrogen-bonded O–H stretching bands [33] and N–H stretching bands [34] that decrease in the presence of HMDS.

**Figure 7:**
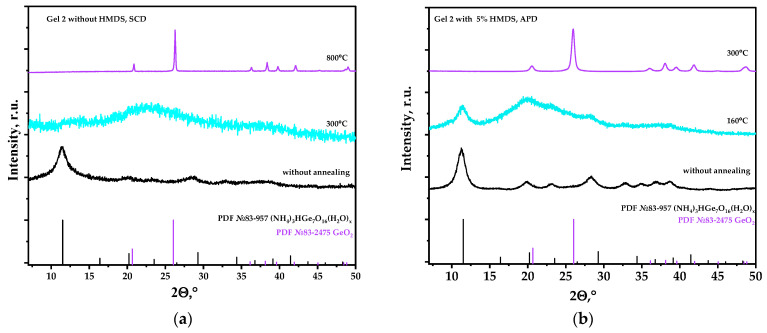
Diffractograms of aerogels (NH_4_)_3_H(Ge_7_O_16_)(H_2_O)_x_ obtained using supercritical drying (**a**) and by drying at atmospheric pressure (with 5 wt.% HMDS) (**b**) after annealing at different temperatures.

The phase composition of aerogels obtained with Gel-2 as precursor, both by supercritical drying and via APD, corresponds to (NH_4_)_3_H(Ge_7_O_16_)(H_2_O)_x_ (Figure 6a). To obtain GeO_2_ aerogels it is necessary to anneal this compound. In the case of aerogels obtained by SCD, annealing at 300 °C led to decomposition of (NH_4_)_3_H(Ge_7_O_16_)(H_2_O)_x_ and formation of X-ray amorphous phase. Germanium dioxide crystallization occurred at a temperature of 800 °C, which was accompanied by complete collapse of the porous structure (Figure 7a) [35]. Annealing of the modified aerogels obtained by APD showed that crystallization of hexagonal germanium dioxide occurs already at 300 °C (Figure 7b). Surface area of the obtained crystalline GeO_2_ aerogel was 50 m^2^/g. This is a great improvement on the SCD method, since SCD does not allow synthesis of crystalline GeO_2_ aerogels with a large surface area. The coherent scattering region of the modified aerogel particles changed from 7 to 20 nm after the annealing.

**Figure 8:**
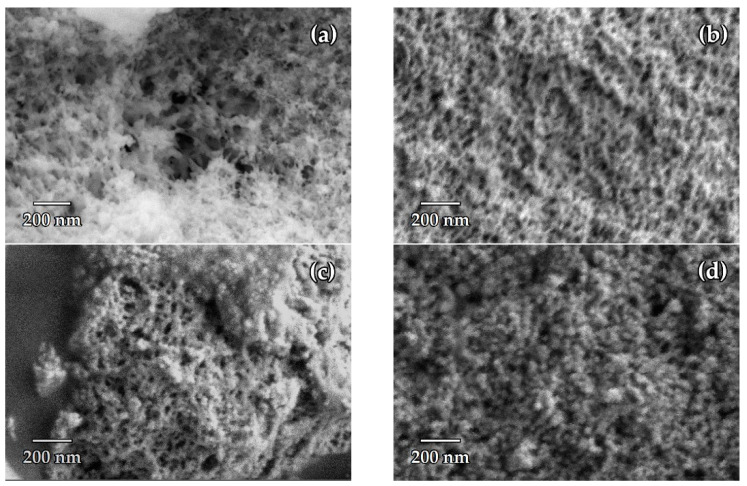
SEM images of aerogel obtained by drying Gel-2 in supercritical CO_2_ after solvent exchange for DMF (**a**); by drying Gel-1 modified with 5 wt.% HMDS at atmospheric pressure (**b**); by annealing of the aerogel obtained by drying Gel-1 modified with 5 wt.% HMDS at atmospheric pressure at 160 °C (**c**) and 300 °C (**d**).

Microphotographs of aerogel obtained by SCD, as well as aerogel obtained by APD and annealed at different temperatures, are given in Figure 8. It can be seen that the 3D network is preserved after annealing 160 °C. The aerogel obtained by APD retains a comparatively large surface area after annealing at 300 °C, but the 3D network is destroyed and the sample consists of nanosized spherical particles.

**Figure 9:**
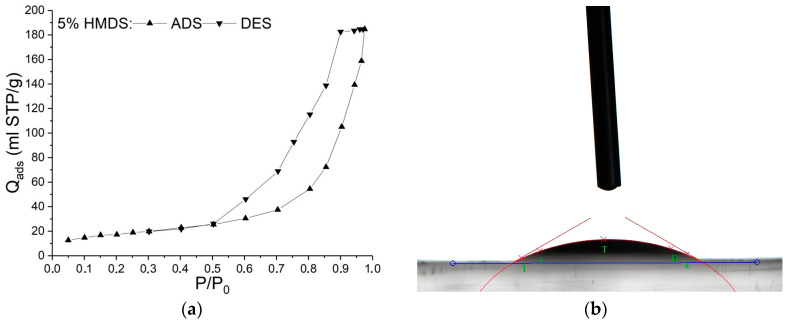
Isotherms of low-temperature adsorption–desorption of nitrogen in aerogel obtained by drying Gel-2 modified with 5 wt.% HMDS at atmospheric pressure (**a**); Photo of a drop on the surface of the aerogel obtained by drying Gel-2 modified with 5 wt.% HMDS at atmospheric pressure, contact angle 30° (**b**).

Nitrogen adsorption–desorption isotherms of aerogel obtained by drying Gel-2 modified with 5 wt.% HMDS at atmospheric pressure belong to Type IV according to IUPAC, which is typical for mesoporous materials, with H1 type hysteresis (Figure 9a). The average pore size was calculated using the Barrett–Joyner–Halenda model and was found to be 23 nm. The cumulative pore volume is 0.290 cm^3^/g.

Both the (NH_4_)_3_H(Ge_7_O_16_)(H_2_O)_x_ aerogel (Gel-2, 5 wt.% HMDS, APD) and the annealed GeO_2_ aerogel sample were found to be hydrophilic, with contact angle of 30°. Without a modifier the samples obtained by SCD had a contact wetting angle of 12° for Gel-2.

## 4. Conclusions

In the present work it was shown that cheap GeO_2_ aerogels with controlled contact angle can be produced by APD. During the work, the most suitable modifiers were selected for gels obtained by different methods in accordance with the type of surface groups, as well as the optimal content of the silylating agent. The largest specific surface area of aerogels obtained by drying at atmospheric pressure was 90 m^2^/g for aerogels modified with MTMS and 140 m^2^/g with HMDS. It was shown that introduction of a modifying agent affects the phase composition and particle size of the aerogel, as well as speed and regularities of thermal decomposition. Employing gels with different preprocessing histories, we were able to produce both hydrophobic and hydrophilic crystalline GeO_2_ aerogels via ambient pressure drying. This inexpensive synthetic route opens up possibilities for GeO_2_ aerogel production for use in LIA, medicinal applications, etc. The possibility to modify the wetting behavior of the aerogel is key to developing functional materials. For example, it has been experimentally shown that the introduction of the active drug substance into the hydrophilic matrix leads to its rapid release [36,37], and release into the hydrophobic one leads to a slow (prolonged) release. That is, the release rate can be adjusted by selecting a suitable modifier [38].

## Figures and Tables

**Table 1:** Comparison of synthesis conditions of the initial gels.

	Gel-1	Gel-2
Precursor	GeCl_4_	GeO_2_
Germanium concentration in the gel	0.3 mmol/mL	0.6 mmol/mL
Solvent	Butyl acetate	Water
pH	Acidic	Basic
Modifier solvent	Heptane	DMF
Phase composition	GeO_2_	(NH_4_)_3_H(Ge_7_O_16_)(H_2_O)_x_

**Table 2:** Comparison of specific surfaces of aerogels obtained by different methods and modified with various organic compounds at 10 wt.% of silylating agent concentration.

Modifier	S_BET_, m^2^/g	
	Gel-1	Gel-2
HMDS	<10	106
MTMS	77	<10
DMCS	<10	<10

**Table 3:** Comparison of specific surfaces of aerogels obtained by different methods with varying concentrations of modifiers.

		Modifier Content, wt.%					
		0 (SCD)	1	2.5	5	10	15
Gel-1	Ge/Si	-	0.5	0.2	0.1	0.05	0.03
	S_BET_, m^2^/g	180	24	62	89	77	<10
Gel-2	Ge/Si	-	2.3	0.9	0.5	0.2	0.15
	S_BET_, m^2^/g	93	75	65	140	106	14

## Data Availability

The original contributions presented in the study are included in the article; further inquiries can be directed to the corresponding author.
